# Valorization of Bio- and Coal-Based Ash in Rigid Polyurethane Foams: Thermomechanical Performance and Decomposition Under Pyrolytic Conditions

**DOI:** 10.3390/ma19204348

**Published:** 2026-10-16

**Authors:** Artur Bukowczan, Patrycja Zakrzewska, Paulina Zając, Tomasz M. Majka, Monika Kuźnia, Krzysztof Pielichowski

**Affiliations:** 1 Department of Chemistry and Technology of Polymers, Faculty of Chemical Engineering and Technology, PK Krakow University of Technology, ul. Warszawska 24, 31-155 Krakow, Poland; paulina.zajac@pk.edu.pl (P.Z.); krzysztof.pielichowski@pk.edu.pl (K.P.); 2 Interdisciplinary Center for Circular Economy, PK Krakow University of Technology, ul. Warszawska 24, 31-155 Krakow, Poland; 3 Department of Heat Engineering and Environment Protection, Faculty of Metals Engineering and Industrial Computer Science, AGH University of Krakow, al. Mickiewicza 30, 30-059 Krakow, Poland; trestka@agh.edu.pl (P.Z.); kuznia@agh.edu.pl (M.K.)

**Keywords:** polyurethanes, sustainability, ash, thermal stability, characteristic of gases

## Abstract

This study investigates the pyrolytic processes and thermomechanical properties of polyurethane (PU) foamed composites filled with ashes obtained after sunflower husk combustion and conventional coal combustion, as well as their silanized derivatives. The results indicate that an improvement in thermal stability during the initial stages of pyrolysis was observed only for PU composites containing silanized ash generated from conventional coal combustion (a difference of 5 °C). At later stages of the thermal decomposition, a significant increase in temperature resistance was observed for the remaining composites. Kinetic studies of degradation using isoconversional methods revealed that composites containing sunflower husk ash exhibit degradation mechanisms different from those of the reference material, as observed by the changes in the activation energy particularly within the conversion range of 0.40–0.65. Dynamic mechanical analysis (DMA) demonstrated that all composites show improved mechanical response over a broad temperature range, with the highest mechanical performance observed after ash silanization (doubled E′ at the initial stage of the study compared to the reference). The analysis of evolved gases, performed using the Py-GC/MS technique, proved the existence of a different degradation path for composites with sunflower husk ash by a change in the amount of evolved gases during the pyrolysis process.

## 1. Introduction

Despite decades of research and a vast number of publications devoted to rigid polyurethane foams (RPUFs), these materials remain an important subject of scientific investigation. This is primarily due to the increasing requirements for modern insulation materials. Achieving low thermal conductivity and enhanced mechanical properties is no longer sufficient as much attention has been paid to fire resistance, durability, long-term stability of properties, reduced environmental impact, and the possibility of managing the material at the end of its service life [1,2,3,4]. In addition, polyurethane materials may provide a potential solution for the accumulation of waste materials, which has become a significant environmental problem. Such waste can be incorporated as a filler into a polyurethane matrix, thereby contributing to the reduction in its accumulation in the environment. Moreover, through appropriate modification, the waste material may act as a functional additive capable of improving selected properties of the polymer itself [5,6,7,8,9,10].

The complex interrelationships between chemical composition, processing conditions, cellular structure, and the functional properties of the resulting foams remain a significant challenge. Improvement of one property often results in deterioration of another, making it difficult to develop a material that simultaneously combines high thermal insulation performance, favorable mechanical properties, and adequate fire resistance [11,12,13]. An additional challenge is the replacement of petrochemical-derived raw materials with renewable alternatives, as well as the development of effective methods for polyurethane recycling. Research into aging phenomena is also becoming increasingly important, since the properties of a foam determined immediately after production do not necessarily reflect the behavior of the material during long-term service. These existing trade-offs, together with emerging technological and environmental requirements, provide a strong rationale for continuing research on these materials [14,15].

In the past few years, researchers demonstrated advanced pyrolysis and multistage thermochemical processes capable of converting real polyurethane mattress waste into gas streams rich in hydrogen, methane and other valuable products, while detailed analytical methods are being used to better control product formation. A particularly promising direction is the development of closed-loop recycling, in which recovered PU components are returned to the production of new polyurethane rather than being used only for energy recovery. Recent studies have demonstrated recycled polyols that can replace a significant fraction of virgin polyol in new PU foams, showing the potential to reduce both waste and the use of fossil-based raw materials [16,17]. Another interesting approach involves the use of state-of-the-art computational techniques, such as ReaxFF, for the molecular modeling of thermal degradation processes [18,19].

The use of ashes derived from the combustion of conventional fuels and biomass as fillers for polymers offers an interesting method for modifying their properties while simultaneously utilizing combustion by-products. Incorporating ash into the polymer structure can influence thermal and mechanical properties as well as other functional properties [20,21,22]. Ashes derived from biomass combustion may exhibit different effects compared to those from conventional fuel combustion, primarily due to differences in their chemical and mineralogical composition. The content of metal oxides and other inorganic constituents in the ashes can modify the thermal decomposition mechanism of the polyurethane matrix, affecting the temperatures at which specific degradation stages occur, and the nature of the volatile products formed. Consequently, the use of different types of ash may lead to either an improvement or a deterioration of specific performance properties of PUR foams, depending on the filler’s characteristics and its interaction with the polymer matrix [23,24,25,26,27].

Silanization offers an additional means of modifying ash properties. Surface modification with silanes can improve the compatibility of ash particles with the polyurethane matrix, reduce particle agglomeration, and enhance interfacial interactions. Consequently, this may lead to a more homogeneous composite structure and alterations in its thermal and mechanical properties. Analysis of the influence of the ash type and its surface modification makes it possible to determine the feasibility of using these materials as functional fillers for PUR foams and to identify potential avenues for their utilization in polymer materials [28,29,30].

The above considerations provided motivation and constituted the principal reason for the current investigation conducted on polyurethane foams containing conventional ashes and ashes derived from the combustion of sunflower husks, as well as their silane-modified counterparts, with the aim of assessing the thermal stability and pyrolysis behavior. The primary focus of the previous research was on combustion processes conducted in a synthetic-air atmosphere [31,32]. Considerably less attention was devoted to investigating the effect of temperature on the behavior of the studied systems under an inert atmosphere. The pyrolytic aspect of these investigations is also highly relevant in the context of flammability studies. Pyrolysis enables the assessment of thermal stability, identification of the compounds evolved, and elucidation of the degradation mechanism that is of primary interest for thermochemical recycling technologies. Numerous researchers have emphasized that pyrolysis itself also occurs during combustion, particularly during its initial stages, and therefore should not be disregarded in comprehensive investigations of the material [33,34,35]. To facilitate the visualization of the effects of incorporating ash into polyurethane foams, the most important properties are summarized in Table 1.

In this study, a series of rigid polyurethane foams modified with ash derived from both conventional and biomass combustion processes was investigated. The influence of ash incorporation on the thermal stability, degradation behavior, and volatile degradation products of the resulting polyurethane systems was evaluated. In addition, the ash fillers were subjected to a silanization treatment to modify their surface properties and assess the effect of this modification on the performance and degradation mechanisms of the PU foams. Attention was paid to the thermal degradation pathways of the investigated materials and to the identification of volatile products released during decomposition. The results obtained provide an insight into the relationship between ash type, surface modification, and the thermal degradation behavior of rigid polyurethane foams that increase the thermochemical recycling potential of this class of sustainable polymeric materials.

## 2. Materials and Methods

### 2.1. Characterization of Filler and Its Modification

The detailed preparation and characterization of the ashes used were described in the previous works [31,32]. Briefly, coal fly ash (C) and sunflower husk ash (B) were used as fillers. The C was sourced from a conventional (pulverized) hard coal-fired boiler at a power plant in southern Poland and collected from electrostatic precipitators. According to the data provided by the manufacturer, the ash meets category A criteria regarding loss on ignition. The sunflower husk ash (B) was obtained via a laboratory combustion process of sunflower husks. This thermal conversion was carried out in an electric furnace for 135 min, a duration optimized to achieve the lowest possible carbon content in the B. In this study, the ashes were incorporated into the polyurethane matrix in both their raw (unmodified) form and after a silanization process (BSi and CSi). To enhance the adhesion between the ash particles and the polyurethane matrix, a silanization process in toluene was applied. Initially, the ash was dried at 105 °C for 15 h to achieve a moisture content of 0.3% and subsequently cooled in a desiccator. The modification procedure began by blending water and toluene using a magnetic stirrer for 15 min. Afterward, [3-(2-aminoethylamino)propyl]trimethoxysilane (C_8_H_22_N_3_O_3_Si) was introduced to undergo hydrolysis for 1 h at 23 °C. The ash was then added to the hydrolyzed silane solution, and the reaction was allowed to proceed for 24 h at 23 °C. To remove any unreacted silane, the treated ash was washed three times with toluene. As a final step, the silanized ash was dried at 105 °C for 15 h.

### 2.2. Preparation of Modified Polyurethane Foams

Rigid polyurethane foams were synthesized using the commercial two-component system EKOPRODUR PM4032 (PCC Group, Brzeg Dolny, Poland). Component A consisted of a polyol system with a blowing agent, fire retardants and catalysts. Component B comprised an isocyanate prepolymer. In accordance with the manufacturer’s specification, components A and B were combined at a weight ratio of 100:110. Firstly, the polyol was mixed with the additive (filler) of raw or silanized coal fly ash or sunflower husk ash (the amount of filler was in each case 10% by mass in relation to the mass of the produced polyurethane composite), and the mixture was stirred until it became completely homogeneous. Based on our previous research results and methodology, we selected a 10 % wt. additive content as having the most beneficial effect on the properties of the foams. In the next step, isocyanate was added to the slurry, and the system was mechanically agitated at 1200 rpm for 8 s. The resulting reactive mixture was cast into a square mold and allowed to cure for 7 days before the samples were demolded and prepared for subsequent testing.

### 2.3. Methods

#### 2.3.1. Infrared Spectroscopy with Fourier Transformation (FTIR)

Fourier transform infrared spectroscopy measurements were performed on a Nicolet iS5 spectrometer (Thermo Scientific, Waltham, MA, USA) equipped with a diamond crystal attenuated total reflectance unit (ATR). Spectra were measured with a resolution of 8 cm^−1^ from 4000 to 400 cm^−1^ with an average of 16 scans.

#### 2.3.2. Thermogravimetry (TG)

Thermogravimetric analysis was performed under a nitrogen atmosphere, using a TG 209 F1 Libra thermogravimetric analyzer (Netzsch, Selb, Germany) with open α-Al_2_O_3_ crucibles with a sample mass of ca. 3 mg. For the kinetic analysis four different heating rates were chosen: 2.5, 5, 10 and 20 K/min.

#### 2.3.3. Pyrolysis-Gas Chromatography/Mass Spectrometry Technique (Py-GC/MS)

The pyrolysis process was conducted in a vertical micro-furnace pyrolyzer PY-3030D (Frontier Lab., Koriyama, Japan) mounted on a Shimadzu GCMS-QP 2010 SE (Shimadzu, Kioto, Japan) under a flow of helium as a carrier gas. To study gaseous products of the thermal decomposition, the flash pyrolysis temperature protocol was set at 190, 320, 390 and 500 °C, and the duration of each pyrolysis step was 12 s. The obtained pyrolyzates (approximately 0.4 mg) were separated using a stainless steel capillary column (Frontier Lab. Ultra ALLOY-5, Koriyama, Japan) coated with 5% diphenyl −95% dimethylpolysiloxane liquid phase following a sequential protocol: 50 °C (1 min hold), 200 °C (1 min hold) and 280 °C (5 min hold), with a heating rate of 40 K/min for the first step, and 30 K/min for the second. The program for the mass spectroscopy detectors was as follows: ion source temperature 200 °C, interface temperature 230 °C, and detector voltage 0.1 kV. The range of the m/z scan was set from 40 to 600. For peak identification, the Frontier Lab. NIST 17 library database was used.

For EGA-MS measurements, the column EGA Tube 2.5 × 0.15 mm I.D. was used. The specific parameters of the EGA column were as follows: furnace temperature 50 °C (0 min)–(20 °C/min)–600 °C; Py-GC ITF temp. 300 °C Auto Mode; GC injection temp. 300 °C, column temp. 250 °C isothermal. The MS detectors were set up according to the following procedure: injection mode: split; carrier gas: Hel; split ratio 1/100; Sample amount ca. 0.5 mg; MS Ion source temp. 250 °C; interface temp. 280 °C, scan range *m*/*z* 29–600, scan time 5s.

#### 2.3.4. Dynamic Mechanical Analysis (DMA)

DMA was performed using a DMA 242 C analyzer (Netzsch, Selb, Germany) in compression mode under a nitrogen atmosphere (50 mL min^−1^). Cubic specimens were cooled with liquid nitrogen and heated from −150 to 200 °C at a rate of 3 K min^−1^. The measurements were carried out at a frequency of 1 Hz, using an oscillation amplitude of 40 µm, a proportional factor (PF) of 1.1, and a maximum dynamic force of 5 N.

#### 2.3.5. Kinetics of Degradation

The kinetic analysis was performed using NeoKinetics software (version 3.5.0.8) by Netzsch. Model-free isoconversional kinetic methods are powerful tools to gain information on the reaction complexity through the activation energy (*E_a_*) vs. α dependency determination. A change in the slope of the *E* vs. *α* plot may generally be associated with a difference in the rate-limiting step of the overall reaction mechanism. Isoconversional methods are based on the principle that states that the reaction rate is only a function of temperature for a given constant value of the extent of conversion.

## 3. Results and Discussion

### 3.1. Thermal Stability

**Figure 1:**
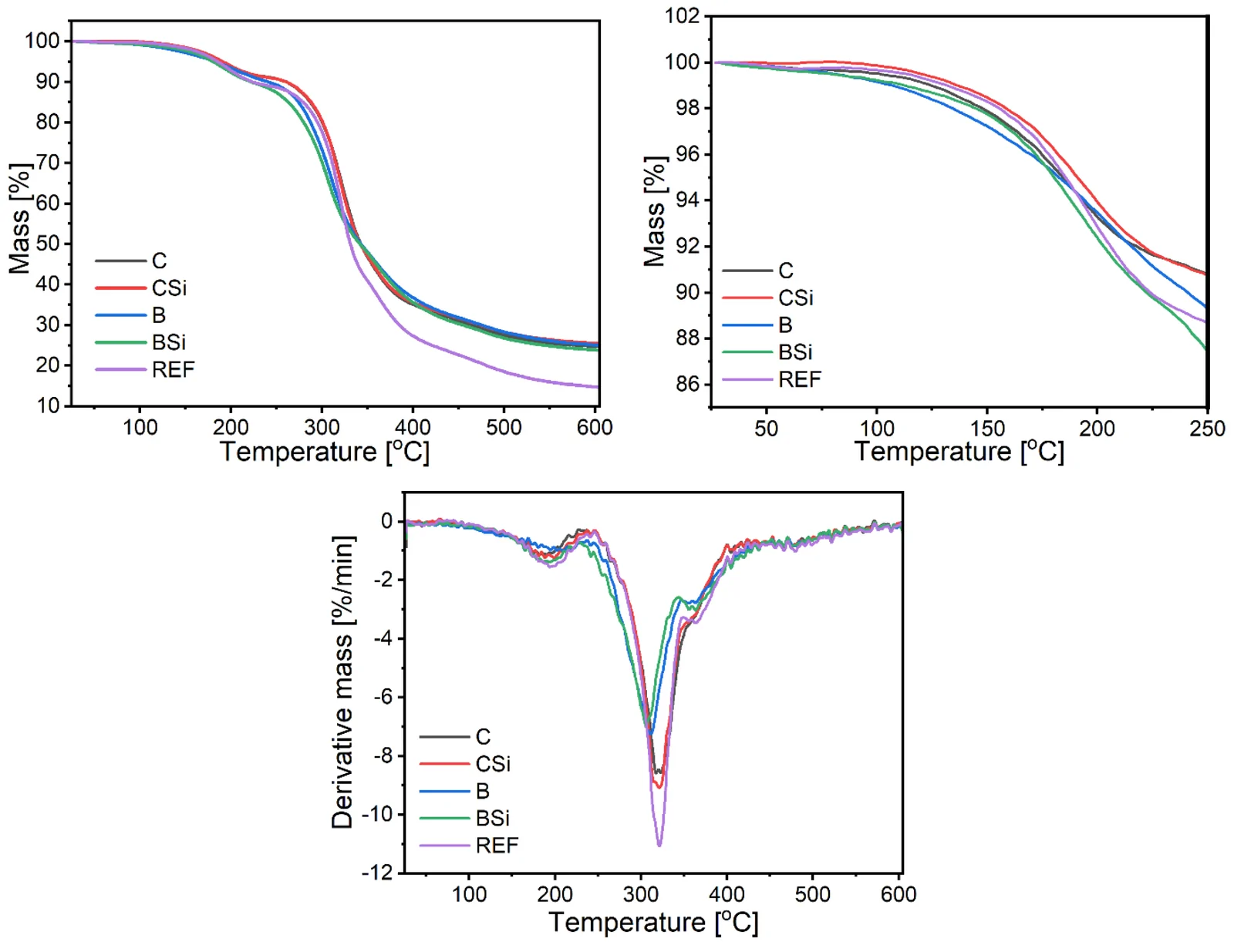
TG and DTG curves of composite polyurethane foams.

The results of the thermal stability of the studied materials are presented in Figure 1, and the characteristic data are collected in Table 2.

The pyrolysis process of polyurethane foams begins with the evaporation of moisture and other volatile components present in the material, corresponding to the first stage of mass loss. As the temperature rises, the thermal decomposition of urethane bonds, the weakest links in the polyurethane chains, gradually occurs; this is observed as the primary decomposition stage, typically associated with a rapid drop in mass. The final observable stage of pyrolytic decomposition is attributed to the breakdown of ether bonds and other related structures derived from the polyol segment. Subsequently, these products may undergo secondary transformations such as cracking, condensation, or reforming which influence the final composition of the gas, liquid, and solid fractions. Ultimately, the process yields pyrolytic gases, liquid products, and a solid carbonaceous residue [37,38]. Analysis of thermogravimetric data indicates varying thermal stability among the tested samples. In terms of the onset temperature of degradation, the system containing silanized conventional combustion ash (CSi) exhibits the highest stability during the initial stage of decomposition, whereas the sample containing sunflower husk ash (B) shows the lowest. This trend persists up to a 10% mass loss.

The difference at the initial stage of degradation between the studied materials may have originated from two mechanisms that can occur: physical shielding and chemical reactions. Physical shielding from degradation may result from the presence of an inorganic component capable of forming a barrier/absorbing some of the energy, thereby slowing down the degradation of the polymer matrix [39,40]. In our case, such an effect can be attributed to the silanization process, in which the addition of a silane compound raises the temperature of initial degradation, especially seen for the system before and after such modification. Another explanation for the differences in thermal stability is the influence of ash composition [36]. In both cases, due to the different origins, the chemical composition differs.

The analysis of ash chemical composition presented in Table 3 reveals significant differences between the various ash types regarding the content of both base metals and heavy metals. It is immediately apparent that ash derived from conventional coal combustion contains a higher proportion of silicates than ash from biomass combustion, a factor that may explain the differences in stability observed among the various composites. Furthermore, biomass-derived ash contains significantly higher levels of calcium, potassium, zinc, and copper; these elements can play a crucial role in catalyzing pyrolysis processes, thereby accelerating degradation [41,42].

Significant changes in thermal decomposition profiles occur during the subsequent stage of the pyrolysis process. Both the addition of conventional coal combustion ash and its modification substantially raise the temperature at which 10% decomposition of the composite takes place. At this stage when the primary decomposition of urethanes occurs, the improvement in thermal stability can be attributed to a shielding mechanism. Interestingly, in the case of composites containing biomass ash, improved stability at this stage of pyrolysis is observed only with the unmodified composition. This suggests a shift in degradation processes, where modification aimed at enhancing compatibility via silanization may yield the opposite of the intended effect for such a system. Analysis of the first derivative of the TG curve confirms an acceleration of the thermal degradation process for the sunflower husk ash system, evidenced by the shift in the main degradation peak toward lower temperatures [43].

**Figure 2:**
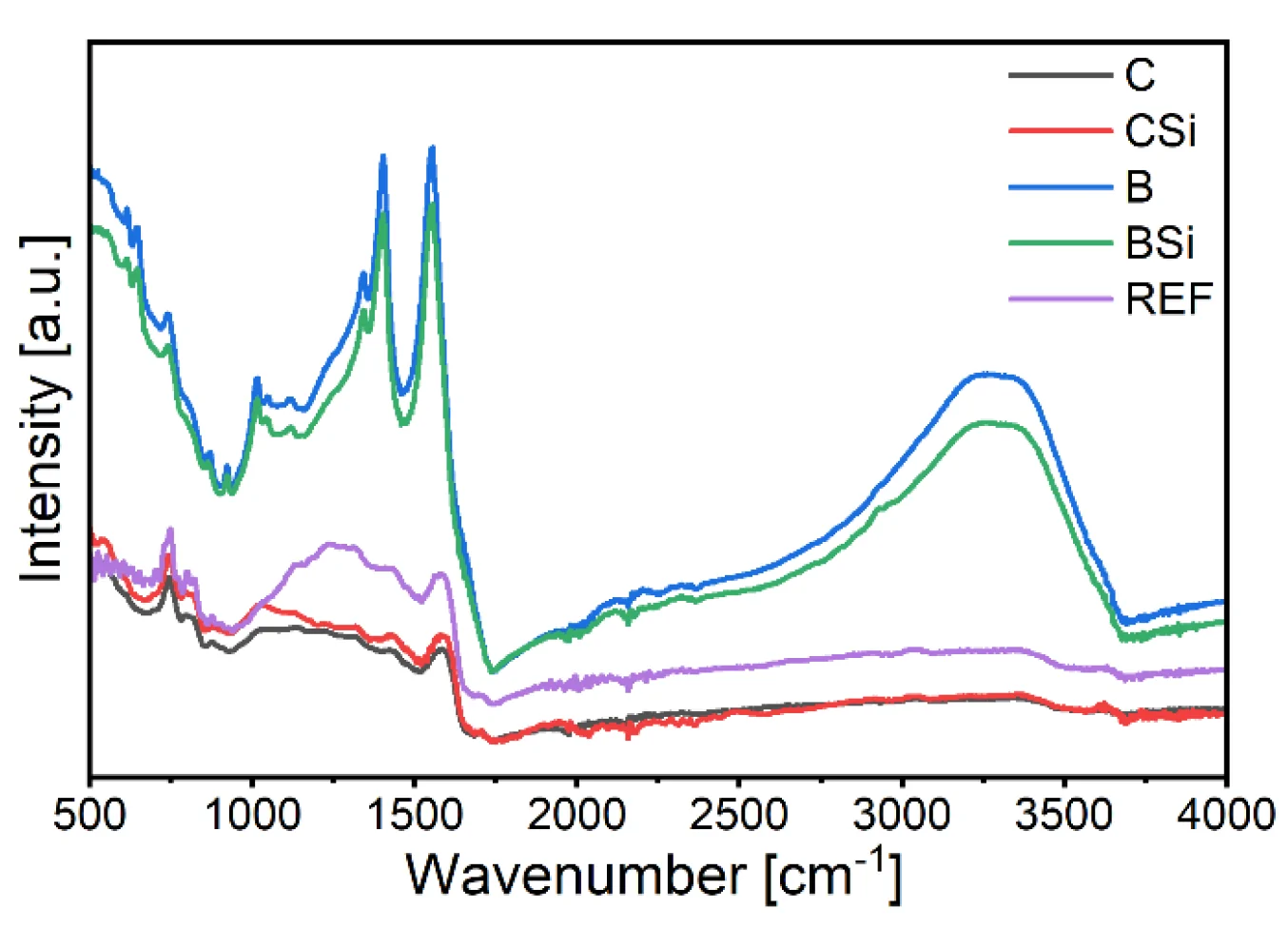
FTIR spectra of solid residue after pyrolysis.

The solid residue from the pyrolysis process indicates that the ash added at an amount of 10 % wt. undergoes no change in mass and is thus not significantly affected by the pyrolytic process. Figure 2 presents the FTIR analysis of the solid residue obtained after thermogravimetric analysis. The spectra of the solid residue from the material containing biomass ash are of particular interest. Bands in the 3000–3500 cm^−1^ range can be attributed to the presence of overlapping hydroxyl or amine groups. Bands at 1402 and 1557 cm^−1^ likely originating from carbonate groups in salts such as calcium or potassium carbonate suggest the presence of a mineral component. This confirms the high mineral content of the ash (as demonstrated in the tests presented in Table 3) as well as its chemical stability, with no changes in its composition or mass following its introduction into and pyrolysis within the PUR matrix. This opens up possibilities for its reuse and aligns with the principles of the circular economy. Interestingly, samples containing ash from conventional coal combustion exhibit an FTIR spectrum very similar to that of pure PUR foam, indicating a very similar chemical nature.

### 3.2. Kinetics of Degradation

**Figure 3:**
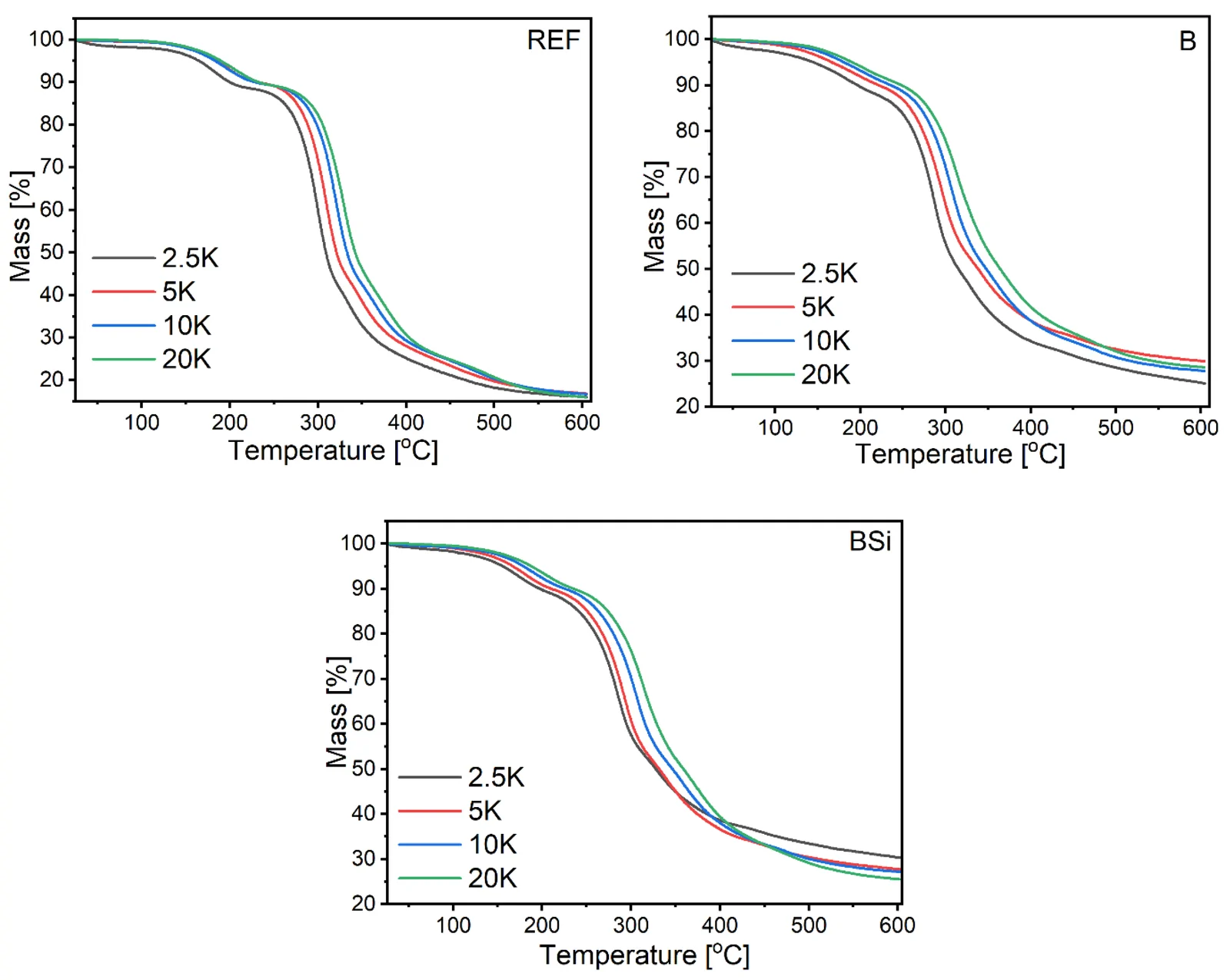
TG data of studied materials recorded at different heating rates.

In order to study the effects of ash addition on PU pyrolysis processes in detail, a study of the kinetics of degradation was performed. As the systems with biomass ash and its silanized derivative differed from the other samples, this section will focus on them. Firstly, the studied samples were subjected to TG measurements at different heating rates (Figure 3), and the data were used in isoconversional methods most often applied for polymer decomposition kinetic studies. The results of fitting are summarized in Table 4.

**Figure 4:**
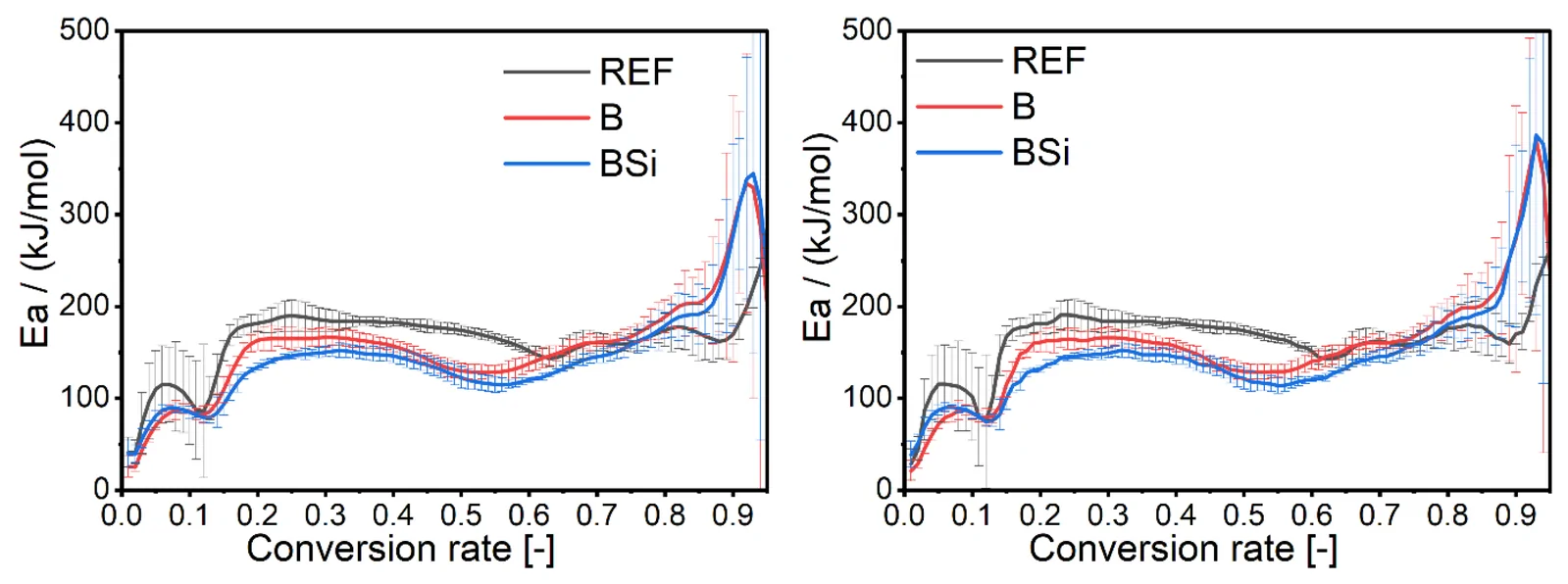
Activation energy vs. conversion rate based on Friedman (**left**) and Vyazovkin (**right**) isoconversion methods. The error bars represent the uncertainty in the determination of the activation energy values for the given conversion degrees.

An F-test can determine whether one model describes the data significantly better than another, or whether the difference in fit could simply result from random variation. The F-statistic is related to the ratio of variances or errors between the models. A low F-value indicates that the models do not differ significantly in terms of fit, whereas a high F-value suggests the difference in fit may be statistically significant. In the degradation kinetic studies, R^2^ (the coefficient of determination) indicates how well a given kinetic model describes the experimental data. Based on the comparison between the tested models, two were chosen for further analysis, namely Friedman and Vyazovkin—Figure 4. The Friedman method is a differential isoconversion method that directly uses the reaction rate to determine the activation energy, while the Vyazovkin calculation method is an integral method and determines the temperature dependencies for different heating rates through numerical optimization, without direct differentiation of the data. As a result, both methods allow for determining the dependence of the activation energy on the degree of conversion without adopting a specific reaction model; however, they may give slightly different values, providing complementary information about the processes under study.

The non-monotonic dependence of activation energy on the degree of conversion indicates the complex nature of the thermal decomposition process in the analyzed systems. The observed changes may be attributed to the overlapping of several degradation stages occurring at different rates and characterized by distinct activation energy values. In particular, the decrease observed at higher conversion levels may signal a shift in the dominant process mechanism. The sharp rise in activation energy at the end of the process may result from the presence of condensed and/or charred fractions in the sample residue that are resistant to pyrolysis and require a higher energy input to undergo decomposition [44,45].

A comparative analysis of the profile progression for the composites using both methods reveals differences between the reference sample and the modified ones, particularly within the 0.16–0.65 conversion range. This suggests the occurrence of distinct thermal degradation mechanisms; the mechanism in the reference sample appears to require higher activation energy, whereas the differences between the various composites are less significant. It can be speculated that the addition of sunflower husk ash influences the degradation process not by limiting or partially absorbing energy transport, but by altering the reaction mechanism itself. The sharp drop in activation energy characteristic of catalytic reactions is not clearly observed, which does not confirm the hypothesis from Section 3.1 regarding the catalytic effect of alkali metals during the initial stages of degradation. The most significant difference in the degradation process between the neat foam and the modified foam is observed within the 0.4–0.6 conversion range. Partial autocatalysis may occur at this stage, as evidenced by a decrease in activation energy. However, it must be noted that at this stage of thermal decomposition, especially in a complex system like polyurethane copolymers, multiple overlapping processes may occur, making such conclusions equivocal. Nevertheless, the effect of the ash addition is observable during the final stage of pyrolysis, at a conversion rate of 0.85. A significant increase in activation energy occurs here, suggesting that the ash promotes the earlier decomposition of more reactive components, leaving behind a more stable material fraction. Additionally, the mineral components of the ash can interact with the organic residue and inhibit its further decomposition, leading to an increase in the apparent activation energy [46,47].

### 3.3. Dynamic Mechanical Analysis (DMA)

**Figure 5:**
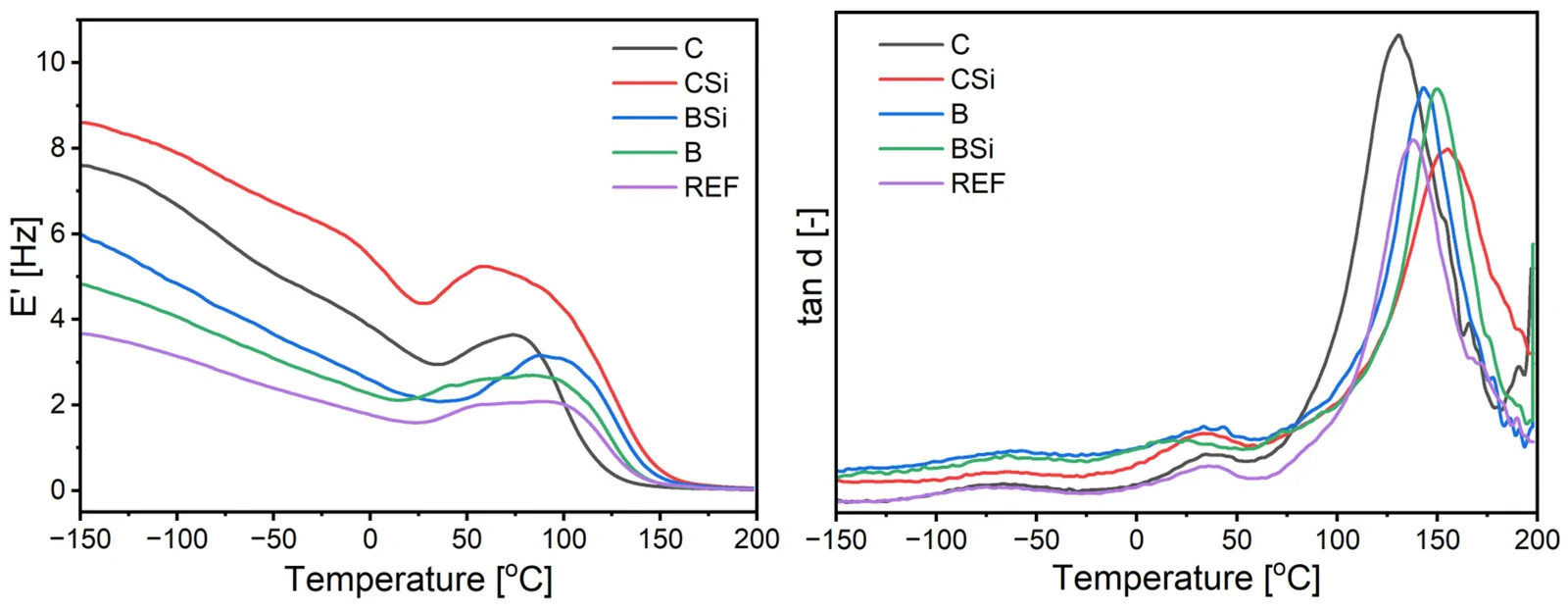
DMA results recorded at 1Hz.

The results of the measurements of mechanical properties upon heating are presented in Figure 5. The glass transition is represented by a drop in the storage modulus E′ which corresponds to a maximum of the loss factor tan d (tan d-peak). For temperatures above ca. 20 °C the foam material is compressed by the oscillating stamp, making the sample “stiffer”, which is reflected in the increase in the storage modulus (E′). When the sample reaches the glass transition temperature, softening of the sample occurs.

Analysis of the material’s mechanical response during heating indicates a reinforcing effect on the polyurethane matrix following the incorporation of 10 % wt.ash. This result may originate from the introduction of inorganic mineral additives into the polymer system, which stiffen the structure and enhance the compressive response across a wide temperature range. Furthermore, samples subjected to additional silanization exhibit a marked improvement in E′ compared to those with unmodified fillers, likely due to the introduction of additional silicate structures into the system. An increase in the storage modulus E′ indicates an increase in material stiffness, which may be associated with improved compressive mechanical performance. These findings demonstrate that, despite reduced thermal stability in certain composite systems upon ash incorporation, other properties can simultaneously be improved [48,49,50].

On the tan δ plot, three effects can be observed: a small, broad peak at approximately −75 °C, which may indicate the onset of local chain segment motions or be associated with β-relaxation. In polyurethane foams, this effect is often linked to the movement of more mobile structural fragments such as segments containing side groups or chain sections with less restricted mobility. A subsequent peak at 30 °C may correspond to the local relaxation of PUR network fragments, for instance, highly mobile segments, less cross-linked regions, or specific parts of soft segments. It is less frequently attributed to α-relaxation (the glass transition), which is more likely to occur near the peak maximum around 150 °C. The addition of ash does not significantly affect the first two observed phenomena. A change is evident in the most pronounced effect, where the ash addition shifts the peak maximum and thus the glass transition temperature toward higher values; this is a well-observed occurrence, particularly when the matrix is reinforced with mineral additives. An exception is the system containing nonsilanized ash from conventional combustion, which lowers the glass transition temperature of the PUR foam. This effect may originate from a localized increase in chain mobility, though its precise nature is difficult to explain unequivocally given the simultaneous increase in E′ [51,52].

### 3.4. Volatile Gas Characterization by Pyrolysis GC/MS

**Figure 6:**
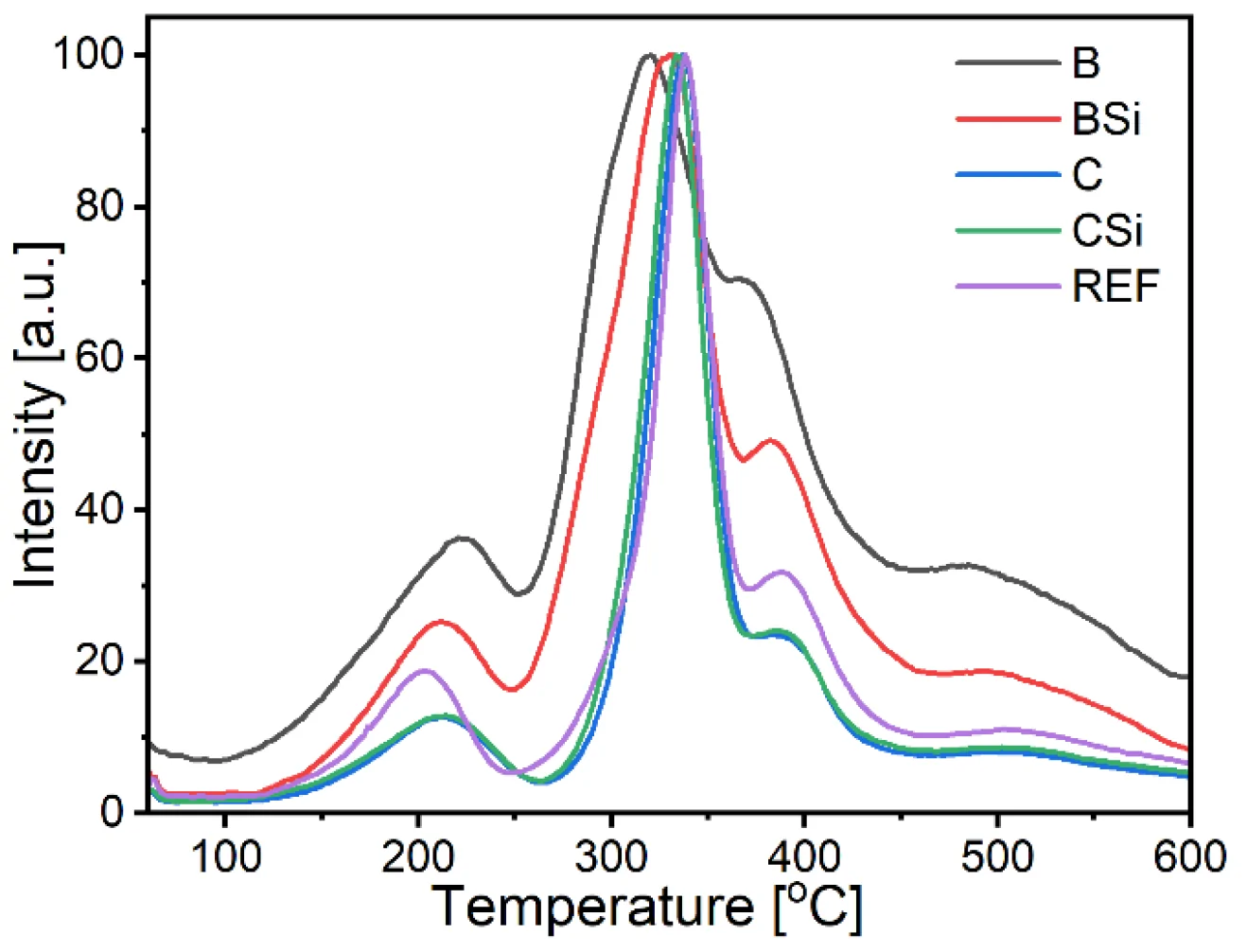
EGA profiles for PU composites.

The detailed analysis of the gaseous products of composite pyrolysis was preceded by Evolved Gas Analysis (EGA)—Figure 6. This technique allows for the characterization of the gas evolution process over a wide temperature range, identifying the regions where the highest quantities of gaseous products are released. This is achieved by monitoring the increased intensity of compounds adsorbed on the column, thereby enabling the identification of specific temperatures optimal for conducting the flash pyrolysis process.

**Figure 7:**
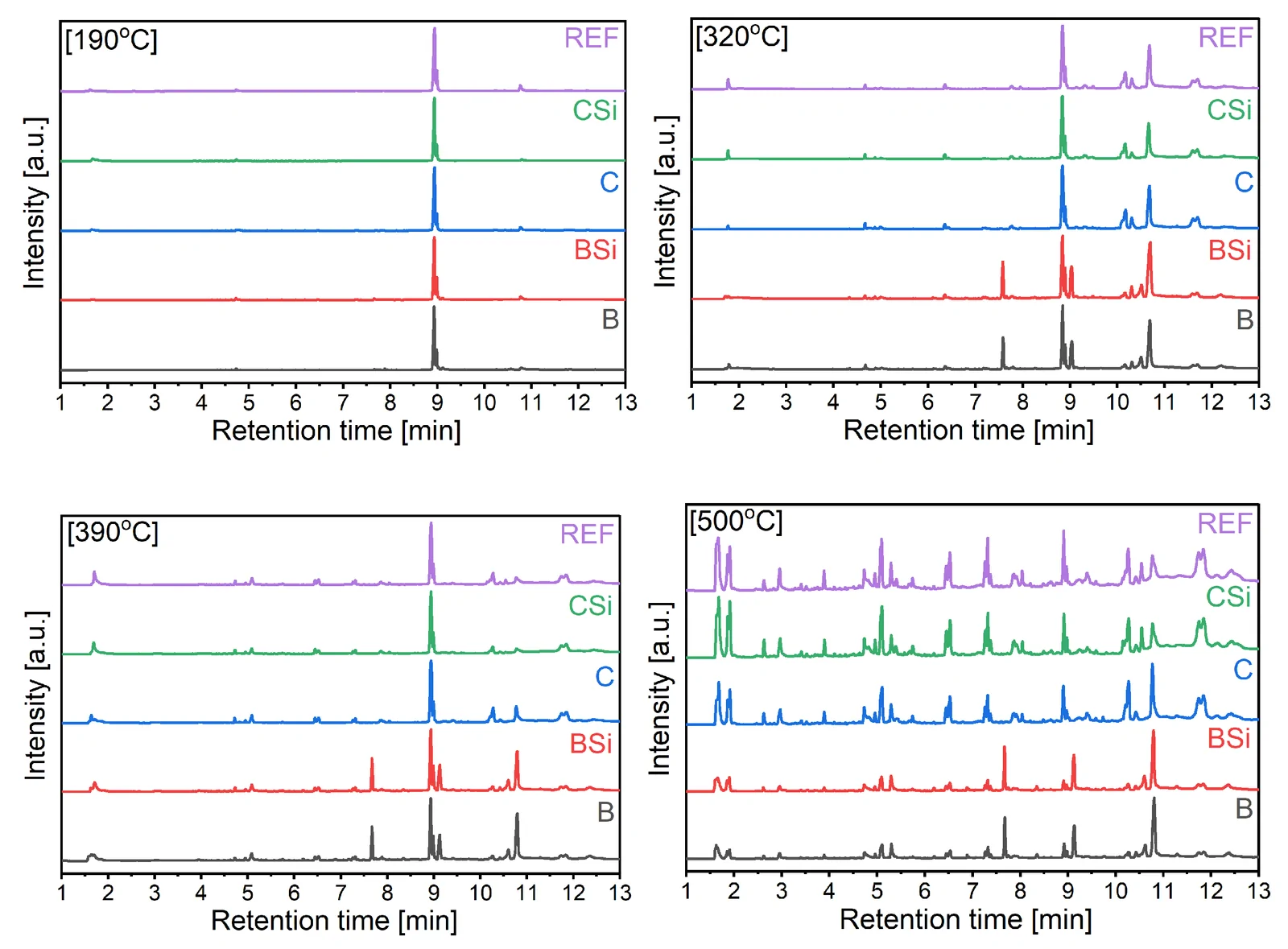
Gas chromatograms of volatile products of studied materials.

Analysis of the profiles in the EGA graph indicates the presence of four main peaks at which the highest gas adsorption occurred on the column, namely, 190, 320, 380, and 500 °C (Figure 7). There were no significant differences in the evolution of gaseous pyrolysis products between the individual systems analyzed. For the composites modified with sunflower husk ash and its modifications, gaseous products adsorbed on the column at lower temperatures, which is consistent with the results observed on DTG, where the peak maximum of mass loss was also lower than in the other systems.

Given that the majority of compounds adsorbed on the column at specific retention times are similar across the materials studied, Table 5 summarizes the identified compounds released during the pyrolysis of the reference foam to ensure clarity in the presentation of results.

Analysis of volatile products of pyrolysis at 190 °C indicates the presence of compounds originating from PUR additives used as flame retardants, catalysts or compatibilizers, such as 2,2-difluoro-propane, N,N-dimethyl- cyclohexanamine, 1-chloro-, phosphate-2-propanol and bis(1-chloro-2-propyl)(3-chloro-1-propyl)phosphate [53]. In this temperature range, the beginning of degradation of the isocyanate component is proven by the presence of 4,4′-methylenebis-benzamine [54]. Among all composite materials, no significant difference at this stage of pyrolysis was observed.

When the pyrolysis temperature rises to 320 °C, the onset of the release of compounds resulting from the decomposition of polyol segments can be observed: 1,1′-oxybis-2-propanol, 2-(2-hydroxypropoxy)- 1-propanol, 2-methyl-2-propanol and products of their initial oxidation: hydroxymethyl 2-hydroxy-2-methylpropionate and dimethyl ester ethanedioic acid [55]. At this temperature, the first differences between the reference sample and sunflower husk ash composites can be observed. For retention time of 7.7 min, the peak ascribed to 2-methyl-2-propanol exhibits a significantly higher intensity than for other samples.

As this compound originates from the decomposition of the main polyol chain, it can be assumed that thermal degradation of these composites proceeds more rapidly and intensively at a pyrolysis temperature of 320 °C. This is supported by both thermogravimetric analysis, which indicates lower thermal stability of these materials, and the degradation kinetics, which suggest a mechanism of decomposition different from that observed for the other materials. Similar effects can be observed during pyrolysis at 390 °C, where the intensity of the peak attributed to the diamine also increases. At this temperature, a carbon dioxide peak with a retention time of 1.7 min can also be observed for all materials, indicating an advanced stage of the degradation process. Pyrolysis at 500 °C results in the evolution of a variety of short-chain compounds originating from the further decomposition of the polyol and isocyanate segments. A significant difference in the intensity of the evolved compounds can be observed for the composites containing ash derived from sunflower husks and its silanized form. In these materials, the intensity of many peaks is markedly lower, which may result from the decomposition of a substantial portion of the polymer chains during the earlier stages of degradation. Consequently, a smaller amount of gaseous products is evolved compared with the other investigated materials. It is also worth noting that the composites containing ash from conventional combustion and its silanized form do not differ significantly from the reference sample. It can therefore be concluded that the addition of this ash does not substantially affect the chemical pathway of thermal decomposition, while its influence on thermal stability is likely to be primarily physical in nature. No silicon-containing compounds were detected, suggesting that silicon and its compounds predominantly remain in the solid residue after pyrolysis.

## 4. Conclusions

The present study investigated the influence of ash additives derived from conventional coal combustion and sunflower husk combustion, as well as their silanized derivatives, on the thermal stability and pyrolysis behavior of rigid polyurethane foams. The results demonstrated that the origin and surface modification of the ash significantly affect the thermal degradation behavior of the PUR matrix. The addition of sunflower husk ash and its silanized derivative resulted in a decrease in thermal stability at the initial stage of degradation. This effect may be attributed to the catalytic activity of the mineral components present in the ash, which can promote the initiation of degradation reactions and accelerate the decomposition of the polymer matrix. However, confirming this hypothesis requires further research as the kinetic calculations do not provide unequivocal evidence for the occurrence of such an effect. In contrast, silanized ash obtained after conventional coal combustion improved the thermal stability of polyurethane foam, most likely due to the formation of a physical barrier by the modified ash particles, which can hinder heat transfer and limit the diffusion of volatile degradation products. Despite the differences observed in the initial thermal stability, all investigated composite materials exhibited an increase in the thermal resistance over a broad temperature range. This indicates that the incorporation of combustion-derived ashes can influence the thermal degradation behavior of PUR beyond the initial stage of decomposition and may contribute to improved resistance under specific thermal conditions. The kinetic analysis of PUR degradation using isoconversional methods showed E vs. α profiles that are due to differences in the degradation mechanism between the samples containing sunflower husk ash and the reference sample. These results suggest that the presence of sunflower husk ash modifies not only the thermal stability of the PUR matrix but also the underlying mechanism of its pyrolytic degradation. The analysis of the composition of gaseous pyrolysis products also revealed distinct differences in the intensity of selected volatile products for the samples containing sunflower husk ash and its silanized form. These observations are consistent with the results of the thermal and kinetic analyses and further confirm that the incorporation of sunflower husk ash affects the course of the PUR pyrolysis process. The observed changes in the gaseous products therefore support the conclusion that the degradation pathway of PUR is altered in the presence of sunflower husk ash and its surface-modified form. The findings presented in our work can be useful for future PU materials recycling technologies, especially those belonging to thermochemical methods that are currently gaining increased attention both in fundamental studies and applied research.

## Figures and Tables

**Table 1:** Summarized properties of PUR/ash composites.

Filler Type	Matrix	Loading (% wt.)	Surface Treatment	Mechanical Performance	Thermal Stability	Pyrolysis/Degradation Observations	Ref.
Coal fly ash	RPUF	5–20%	None	Increased compressive strength and Young modulus up to 20 % wt. load	Increased thermal stability	T_5_% ~170–190 °C,	[31]
Sunflower husk ash	RPUF	3–10%	None	Hardness decreases; friability increases	Improved stability up to ~250 °C	T_5_%: 172 °C at 10 % wt.; T_50_%: 374 °C	[32]
Biomass bottom ash	RPUF	5%	None		Decreased flammability	Residue at 600 °C: 16.98–19.03% for individual ashes	[24]
Pine/spruce/ oak/birch/ paper ash	PU/PIR	1–9%	None	Pine ash 9 % wt.: From 0.11 to 0.36 MPa compressive strength	Thermal stability increased for pine ash and oak ash	Residue at Temp. 1000 °C: reference foam 5%, range for ash-containing foams: 3.1% (oak ash) to 17% (pine ash)	[25]
Fly ash	RPUF	5–20%	None	up to +21% vs. silica compressive strength	-	-	[23]
Rice husk ash	Tung-oil-based viscoelastic PU	1–5%	None	Strength/modulus decreased with loading	Unaffected	Higher solid residue	[36]
Fly ash	PU	0.5, 1.5, 2.5, and 3.5%	Silanization	Compression + 21.6%; toughness + 10.1%	-	-	[28]

**Table 2:** Thermogravimetric data.

	3% [°C]	5% [°C]	10% [°C]	50% [°C]	DTG_max1_ [°C]	DTG_max2_ [°C]	Residue [%]
REF	168.76	185.26	223.76	331.26	195.76	321.76	14.75
C	162.26	184.26	260.76	342.26	190.26	321.76	25.79
CSi	172.76	190.76	261.26	342.26	194.26	322.26	25.12
B	153.76	179.96	240.96	343.26	200.26	311.76	25.03
BSi	161.26	180.76	223.26	342.26	191.26	306.26	23.87

**Table 3:** Metallic element content and chemical composition of the investigated ashes [31,32].

Filler	Metallic Element Content, mg/kg										
	Zn	Cu	Pb	Ni	Cr	Co	Mn	V	Ba	Mo	B
C	139.0	43.7	84.8	62.7	73.4	23.7	281.0	138.0	895.0	5.4	210.0
B	678.0	323.0	2.8	69.3	574.0	4.5	334.0	2.3	53.9	8.2	498.0
Filler	Chemical Composition, %										
	K	Ca	Mg	S	P	Si		Al	Fe		Cr
C	9.4	6.4	0.7	1.00	0.8	35.5		18.1	21.3		0.08
B	34.66	26.18	2.71	2.94	1.28	0.51		0.17	1.74		0.46

**Table 4:** Results of fitting TG data to isoconversion methods.

		Friedman		Ozawa–Flynn–Wall		Vyazovkin		Kissinger–Akahira–Sunose	
Sample		R^2^	F-Test	R^2^	F-Test	R^2^	F-Test	R^2^	F-Test
REF		0.99987	1.162	0.99384	54.701	0.99988	1.041	0.99281	54.710
B		0.99966	1.334	0.99383	24.079	0.99969	1.231	0.99202	31.148
BSi		0.99975	1.161	0.99079	42.447	0.99978	1.024	0.99154	38.987

**Table 5:** Volatile products of pyrolysis detected for polyurethane foams.

Ret. Time	Compound
**190 °C**	
1.62	2,2-difluoro- propane
4.72	N,N-dimethyl- cyclohexanamine
8.94	1-chloro-, phosphate-2-propanol
8.99	Bis(1-chloro-2-propyl)(3-chloro-1-propyl)phosphate
10.76	4,4′-methylenebis- benzenamine
**320 °C**	
1.76	2,2-difluoro- propane
4.66	N,N-dimethyl- cyclohexanamine
4.88	1,1′-oxybis-2-propanol
5.02	2-(2-hydroxypropoxy)- 1-propanol
6.43	Hydroxymethyl 2-hydroxy-2-methylpropionate
7.77	2-methyl-2-propanol
8.84	1-chloro-, phosphate-2-propanol
8.90	Bis(1-chloro-2-propyl)(3-chloro-1-propyl)phosphate
9.30	Hydroxymethyl 2-hydroxy-2-methylpropionate
9.48	2-(2,4,6-Trinitrophenyl)-3-oxobutanoic acid,ethyl ester
10.30	4,4′-methylenebis- benzenamine
11.69	Dimethyl ester ethanedioic acid
12.26	3-pentanol
**390 °C**	
1.70	Carbon dioxide
4.73	N,N-dimethyl- cyclohexanamine
5.09	1,1′-oxybis-2-propanol
6.44	Hydroxymethyl 2-hydroxy-2-methylpropionate
7.86	2-methyl-2-propanol
8.94	1-chloro-, phosphate-2-propanol
10.27	2-methyl-2-propanol
10.54	1,1′-methylenebis[4-isocyanato-benzene]
10.77	4,4′-methylenebis- benzenamine
11.74	2-methyl-2-propanol
**500 °C**	
1.66	Carbon dioxide
1.91	Allyl chloride
2.62	L-Lactic acid
2.95	(S)-(+)-1,2-propanediol
4.73	Aniline
4.95	1,1′-oxybis-2-propanol
5.09	2,2′-oxybis-1-propanol
5.29	p-Aminotoluene
5.40	2-butyl-1-methyl-pyrrolidine
6.45	Hydroxymethyl 2-hydroxy-2-methylpropionate
6.52	2-methyl-1-propanol
7.86	2-methyl-2-propanol
8.91	1-chloro-, phosphate-2-propanol
8.97	Bis(1-chloro-2-propyl)(3-chloro-1-propyl)phosphate
9.39	Dimethyl ester ethanedioic acid
10.42	2-methyl-2-propanol
10.54	1,1′-methylenebis[4-isocyanato-benzene]
10.77	4,4′-methylenebis- benzenamine
11.74	2-methyl-2-propanol

## Data Availability

The original contributions presented in this study are included in the article. Further inquiries can be directed to the corresponding authors.
